# Assessment of Course-Based Research Modules Based on Faculty Research in Introductory Biology

**DOI:** 10.1128/jmbe.00148-21

**Published:** 2021-07-30

**Authors:** Megan F. Cole, Meleah A. Hickman, Levi Morran, Christopher W. Beck

**Affiliations:** a Department of Biology, Emory University, Atlanta, Georgia, USA

**Keywords:** course-based research experience, short-term research experience, modules, self-efficacy, science identity, student affect, research-teaching collaboration

## Abstract

Calls for early exposure of all undergraduates to research have led to the increased use and study of course-based research experiences (CREs). CREs have been shown to increase measures of persistence in the sciences, such as science identity, scientific self-efficacy, project ownership, scientific community values, and networking. However, implementing CREs can be challenging and resource-intensive. These barriers may be partly mitigated by the use of short-term CRE modules rather than semester- or year-long projects. One study has shown that a CRE module captures some of the known benefits of CREs as measured by the Persistence in the Sciences (PITS) survey. Here, we used this same survey to assess outcomes for introductory biology students who completed a semester of modular CREs based on faculty research at an R1 university. The results indicated levels of self-efficacy, science community values, and science identity similar to those previously reported for students in the Science Education Alliance-Phage Hunters Advancing Genomics and Evolutionary Science (SEA-PHAGES) full-semester CRE. Scores for project ownership (content) were between previously reported traditional lab and CRE scores, while project ownership (emotion) and networking were similar to those of traditional labs. Our results suggest that modular CREs can lead to significant gains in student affect measures that have been linked to persistence in the sciences in other studies. Although gains were not as great in all measures as with a semester-long CRE, implementation of modular CREs may be more feasible and offers the added benefits of exposing students to diverse research fields and lab techniques.

## INTRODUCTION

Multiple calls have been made to provide early exposure of undergraduate students to research experiences in order to increase retention in STEM (science, technology, engineering, and math) fields (1–3). Research experiences for undergraduates have been tied to various benefits for students, such as increased interest in research careers and improved research-related skills (4–8). Traditionally, undergraduate research experiences have been through apprenticed work in a research laboratory either during the academic year or over the summers. These experiences have been shown to benefit students but are not feasible for all students due to space and financial constraints on their offering as well as the lack of faculty research on some campuses. To more broadly reach undergraduate students, some schools have transformed undergraduate laboratory classes into research experiences. These courses, termed course-based research experiences (CREs), have been shown to offer many of the same benefits of mentored research but on a broader, more accessible scale (2). For this reason, many institutions have been developing and implementing CREs for their students. However, CREs can follow a number of implementation models, and the impacts of these formats have not yet been fully explored.

One aspect of CREs that may impact student outcomes is whether the research is connected to a national project or developed locally from faculty research at their institution. National projects have the benefit of connecting students to a broader research community and built-in network. Examples of national CREs include the Tiny Earth Initiative (https://tinyearth.wisc.edu) (9), the Small World Initiative (http://www.smallworldinitiative.org), the Genomic Education Partnership (GEP) (10), and Science Education Alliance-Phage Hunters Advancing Genomics and Evolutionary Science (SEA-PHAGES) (11). In these projects, students from a number of diverse institutions and global locations collect data according to standardized experimental protocols and report findings back to a shared database. Often, students have opportunities to interact with the broader research community of the CRE via poster sessions, symposiums, or Web events.

Conversely, locally developed CREs have the benefits of integrating faculty research and teaching and of connecting students to the research community at their own institution. Many institutions have developed CREs based on faculty research, with the largest example of locally developed CREs being the Freshman Research Initiative (FRI) at the University of Texas—Austin, where faculty develop and run research streams for undergraduates (12). Local CREs provide an opportunity for faculty to bridge their research and teaching responsibilities, perhaps alleviating tension between these two professional identities (13). Partnerships between educators and research scientists to develop CREs may also help reconcile a disconnect in how these two groups view laboratory courses. Local CREs also create a unique pipeline to involve undergraduates in apprenticed research at their home institution through exposure to faculty projects that may be immediately accessible to them.

Another important aspect of CRE models that may impact students’ experience is the length of the research experience, which can vary from a single lab period to multiple semesters. While most published CREs are conducted over the course of a full semester or even multiple semesters, short-term research experiences (SREs) are conducted with other lab projects during a semester course. The Prevalence of Antibiotic Resistance in the Environment (PARE) project is one example of a national SRE that has been implemented in dozens of institutions and uses 3 to 4 class periods in a semester (14). SREs such as PARE can be implemented in the context of a traditional laboratory or with other SREs.

CRE length in relation to learning gains has been considered in only a few studies. In a comparison of student self-reported gains in 49 courses across seven different institutions, Mader et al. (15) found greater gains for students in semester-long CREs than in modular CREs. Similarly, gains for students who participated in the San Diego Biodiversity Project month-long modular SRE in a traditional laboratory context were not as great as with a semester-long CRE (16). Longer-term CREs may allow deeper development of project ownership in students or provide more opportunities for failure and iteration, both of which have been tied to beneficial student outcomes (17). However, modular CREs in which students are involved in experimental design and the research outcomes are novel may result in gains similar to those with semester-long CREs (15). In addition, SREs may lower implementation barriers as they can be developed and implemented without needing to overhaul a full-semester curriculum all at once.

One model for the use of SREs is to utilize multiple SREs to fill a semester course, which we term modular CREs. This model may capture outcomes similar to those with semester-long CREs while also offering some unique benefits. Modular CREs can expose students to a wider array of research systems and broad areas when the SREs used are diverse. Also, modular CREs can involve more local research scientists in curriculum development and expose students to more immediately accessible research opportunities.

Here, we examine the use of a local modular CRE model in a semester of introductory biology labs for undergraduates at an R1 university. The modular CRE includes three distinct SREs and introduces students to a range of research approaches and faculty research from the local scientific community. The modular CRE was implemented in the second semester of the introductory biology series for science majors and was assessed with two survey instruments.

In particular, we used the two surveys to address the following research questions: (i) do modular SREs contribute to students’ perceptions of their learning related to basic experimentation skills in biology, and (ii) do modular SREs result in measures of student affect similar to those with a semester-long CRE? Based on the results of previous comparisons between modular CREs and SREs (15, 16), we hypothesized that students in our course would have outcomes similar to those of students in semester-long CREs, especially because our series of SREs encompassed the entire semester and involved students examining novel research questions. To explore the first research question, we developed and administered a student survey to evaluate students’ perceptions of each of the three modular SREs with respect to experimentation skills (18). To examine the second research question, we used the Persistence in the Sciences (PITS) survey instrument (19) as it would allow us to identify specific psychosocial measures that may be affected to a greater or lesser extent in modular CREs than in semester-long CREs, which could aid in further improving the modular CRE design. We used the PITS survey to measure changes in students’ self-efficacy, science identity, and science community values as well as their perceptions of ownership and networking and compared the outcomes for our modular CRE model to outcomes for the SEA-PHAGES project (20), which is a semester-long, national CRE. While the PITS survey measures only short-term outcomes for students, these measures have been shown to be correlated with longer-term gains in outcomes such as persistence (19).

## METHODS

### Biosafety and IRB

All students were trained in biosafety on the first day of the semester, and all appropriate biosafety protocols associated with microbes were maintained for all three modules. Our human subject research was approved by Emory University’s Institutional Review Board (IRB) (approval number 00106478).

### Course context

We performed our study at Emory University, a highly selective research university with approximately 6,000 undergraduate students. Participants were students enrolled in the Foundations of Modern Biology II Lab in the spring of 2018. The laboratory is a 2-credit-hour course with a co-requisite of the Foundations of Modern Biology II 3-credit-hour lecture course. Students are predominantly freshmen (65%) and sophomores (30%). In spring 2018, 555 students were enrolled in the course. Labs met once a week for 3 hours throughout the semester, with at most 24 students in a lab section. Sections were taught by graduate or postdoctoral instructors with an undergraduate learning assistant, and students worked in collaborative groups of 4 on lab projects. A total of 24 unique lab instructors each taught all three modules to the same student section all semester.

### Modules

The lab course was comprised of three modules based on faculty research, which are briefly summarized here. More detailed descriptions can be found in Appendix S1 in the supplemental material. In a 5-lab-period module developed by Christopher W. Beck, students investigated whether the diet or sex of bean beetles (Callosobruchus maculatus) affected their microbiome (21), as a part of a larger research project to understand factors that influence the gut microbial communities of bean beetles (22). In a 2-lab-period module developed by Meleah A. Hickman, students investigated whether the ploidy status of Candida albicans affected mutation rates. In a module developed by Levi Morran, students evolved a population of Caenorhabditis elegans over the course of the semester in the presence of a pathogenic bacterium.

### Survey instrument

Our survey instrument consisted of a set of Likert scale questions that we developed that were aimed at assessing students’ perceptions of each of the modules’ contribution to their learning gains related to experimentation, the PITS survey (19), and a series of demographic questions. To measure students’ perceptions of the modules’ contribution to their learning gains related to experimentation, we asked them to rate how much they felt a module and its associated assignments aided their learning in each of seven areas. The seven areas were based on basic competencies of biological experimentation (18) and included understanding the primary literature, developing a research question, experimental design, conducting experiments, analyzing results, drawing conclusions, and communication. A scale from 1 (not at all) to 4 (to a great extent) was used for these questions. The PITS survey (19) consists of a total of 39 Likert scale questions associated with six scales (Appendix S4). The survey is amenable to assessment on the time scale of a single semester or year, and the scales are correlated with longer-term outcomes such as persistence in STEM (19). The survey is typically administered just at the end of the semester (e.g., see reference 20) because three of the scales (project ownership content, project ownership emotion, and networking) relate to the activities of students during the semester. However, the other three scales (self-efficacy, science identity, and scientific community values) are measures of student affect at any point in time. Therefore, we examined self-efficacy, science identity, and scientific community values in a pre-semester/post-semester format but examined the other scales only at the end of the semester. Demographic questions consisted of self-identified race/ethnicity and gender questions, first-generation status, and expected course grade.

### Survey administration

We administered our survey to students enrolled in the second-semester lab portion of introductory biology at Emory University. Students participated in the survey online as an assignment worth roughly 1% of their overall grade for the semester. Student responses were anonymous, and an alternative assignment was offered for students who did not wish to participate in the study (IRB approval number 00106478). A pre-semester survey consisting of only the measures of self-efficacy, science identity, and scientific community values along with information on previous coursework and year was delivered in the first 2 weeks of the course, and the full survey along with demographic questions were delivered in the last 2 weeks of the course.

### Data analysis

We validated the PITS instrument on our student population by measuring the internal consistency of each scale using Cronbach’s alpha value and with confirmatory factor analysis using the lavaan package in R (23). Detailed information on survey validation can be found in Appendix S2.

To determine whether students’ self-efficacy, science identity, and science community values changed from the beginning of the semester to the end of the semester, we used paired *t* tests. In addition, we estimated effect sizes using Cohen’s *d*, controlling for the correlation between pre-semester and post-semester scores.

To compare our student outcomes on the PITS with those of a traditional lab, or a semester-long CRE, we used data reported previously by Hanauer et al. (20) for students who participated in the SEA-PHAGES project (15, 20). For both comparisons, we conducted two-sample *t* tests with unequal variances.

## RESULTS

### Student population

Of the 555 students enrolled in the course, 507 completed the pre-semester survey (91% participation), and 517 completed the post-semester survey (93% participation). Participants were predominantly first- and second-year students (63% and 31%, respectively), with a higher proportion of females than males (62% and 38%, respectively) (Table 1). The population had 23% PEERs (persons excluded due to ethnicity or race) (24) and 21% first-generation college students.

**TABLE 1:** Demographic information on students in the study

Demographic variable^*a*^	Sample size [no. (%) of students]
Race/ethnicity	
PEERs	115 (22)
Not PEERs	384 (74)
Not reported	18 (3)

Gender	
Female	313 (61)
Male	191 (37)
Not reported or other	13 (3)

First-generation university student	
Yes	109 (21)
No	404 (78)
Not reported	4 (1)

Yr^*b*^	
Freshman	311 (63)
Sophomore	153 (31)
Junior	24 (5)
Senior	2 (<1)

a PEERs, persons excluded due to ethnicity or race (24).

b From pre-semester survey data.

### Student perceptions of modules

**FIG 1:**
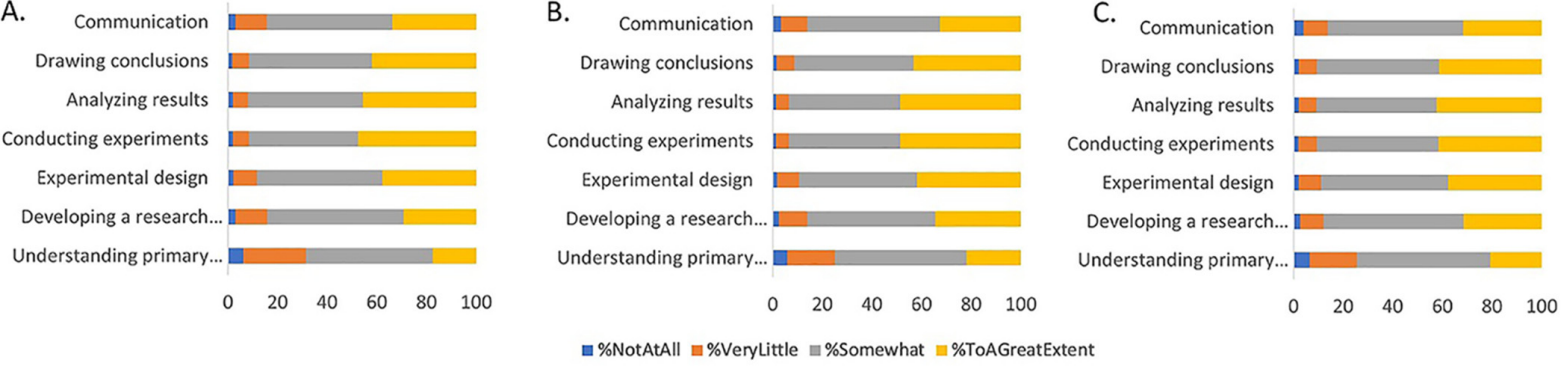
Students rate diverse laboratory modules similarly for how well they aided their learning. Students were asked to rate each lab module (C. elegans evolution [A], bean beetle microbiome [B], and yeast mutation rate [C]) for how much it aided their learning in seven different broad skills (understanding the primary literature, developing a research question, experimental design, conducting experiments, analyzing results, drawing conclusions, and communication) on a scale of 1 (not at all), 2 (very little), 3 (somewhat), and 4 (to a great extent). The sample size was 448 for all modules.

As the three lab SRE modules vary in their model systems, numbers of lab class periods devoted to them, lab techniques utilized, analyses conducted, and the levels of uniqueness expected for each group’s data, we explored whether students perceived differences in how each module contributed to their learning related to experimentation. Students rated all three modules similarly in terms of how much they contributed to their learning across the seven learning objectives that we queried (Fig. 1). For all learning objectives, the majority of students reported that the modules aided their learning “somewhat” or “to a great extent.” Across all modules, students reported learning the most about conducting experiments and analyzing results and learning the least about understanding the primary literature. The largest difference was seen in the objective of understanding scientific literature, where the average rating for the beetle microbiome module was 0.11 points higher than the average rating for the C. elegans evolution module (*t* = 2.14; df = 894; *P* = 0.03 [by a paired *t* test]).

### Gains in measures of persistence in the sciences

**FIG 2:**
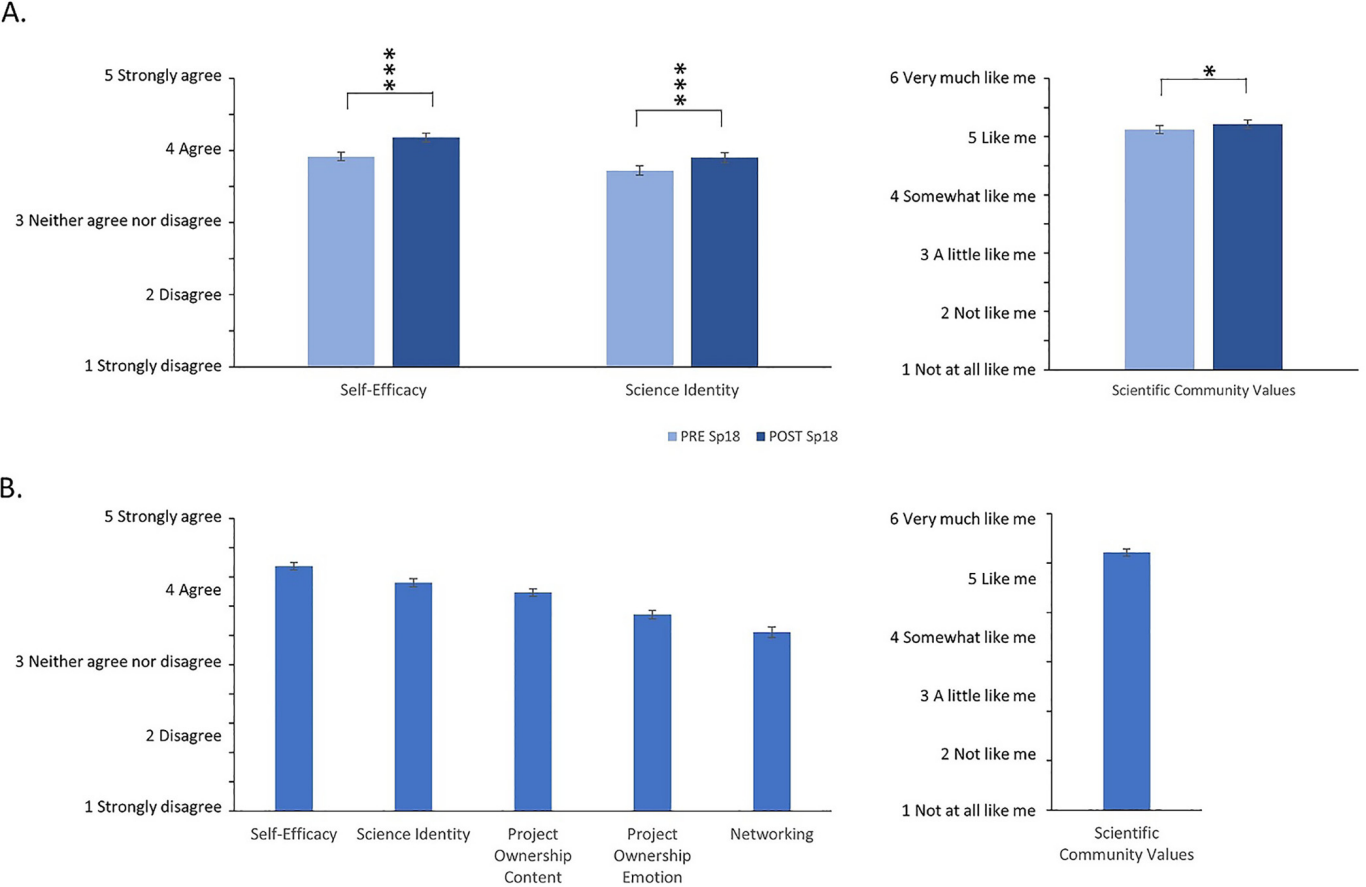
Students in modular CREs show gains in measures of persistence in the sciences. (A) Average scores and 95% confidence intervals for students in a modular CRE course at Emory. Scores for self-efficacy and science identity used a 5-point scale, while scientific community values used a 6-point scale. *P* values were calculated using paired *t* tests (* indicates a *P* value of <0.05, ** indicates a *P* value of <0.01, and *** indicates a *P* value of <0.001). (B) Post-semester scores for all six measures. Scientific community values were measured on a 6-point scale, while all other measures used a 5-point scale.

From the beginning of the semester to the end of the semester, students showed significant gains in self-efficacy (*n* = 450; *t* = −7.48; *P* < 0.001), science identity (*n* = 448; *t* = −5.39; *P* < 0.001), and scientific community values (*n* = 444; *t* = −2.34; *P* = 0.02) (Fig. 2A). However, the effect sizes were low for all measures of student affect (self-efficacy, 0.352; science identity, 0.255), particularly scientific community values (0.109).

In addition to self-efficacy, science identity, and scientific community values, measures of project ownership and networking were assessed in the post-semester survey only as these measures relate to the students’ experiences with the laboratory projects carried out in the course (Fig. 2B). Scores were relatively high for all categories, with the highest average ranking for scientific community values (5.2 on a 6-point Likert scale) and the lowest relative ranking for networking (3.1 on a 5-point Likert scale). Individual item averages within each category varied (see Appendix S3 in the supplemental material), with the greatest differences observed within networking, where students ranked “I have discussed the research in this course with my friends” 0.8 points higher than “I have discussed the research in this course with my parents,” and within project ownership content, where the average score for “I was responsible for the outcome of my research” scored 0.5 points higher than “I had a personal reason for choosing the research project I worked on.”

### Students in modular CREs compared to semester-long research experiences

**FIG 3:**
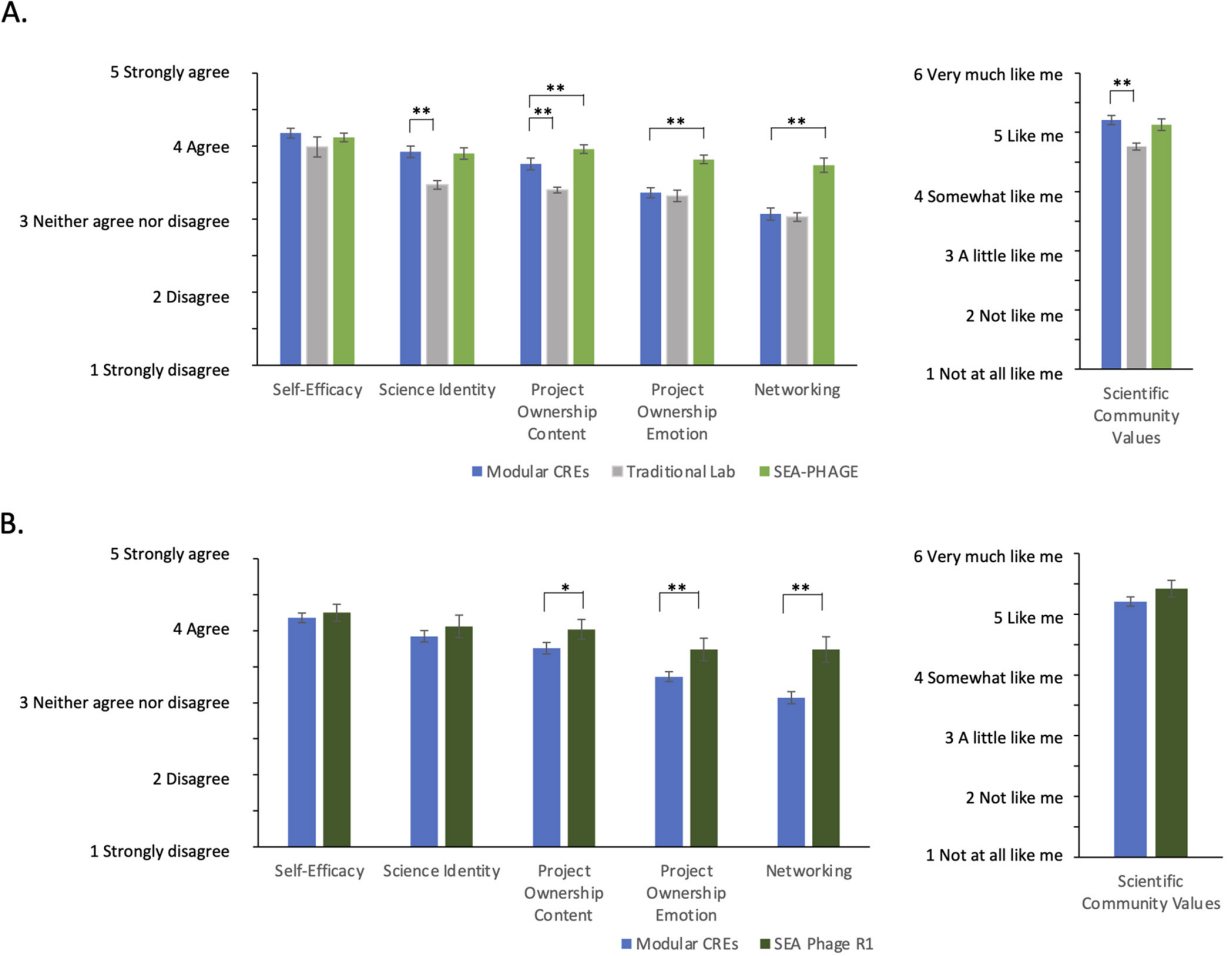
Students in modular CREs score as well as semester-long CRE students in some measures of persistence in the sciences. Average scores and 95% confidence intervals are shown for students in a modular CRE course at Emory (*n* = 517), a traditional lab course (*n* = 1094) (data from reference 20), and the SEA-PHAGES CURE (*n* = 335) (data from reference 20) (A) and students in a modular CRE course at Emory (*n* = 517) and the SEA-PHAGES CURE at R1 institutions (*n* = 100) (data from reference 20) (B). Scores for scientific community values used a 6-point scale but were normalized to a 5-point scale here. *P* values were calculated using two-sample t tests (* indicates a *P* value of <0.01, and ** indicates a *P* value of <0.001).

To assess how our modular CRE model compared to traditional lab and semester-long CRE courses, we compared our results to those of Hanauer et al. (20), who surveyed students in a variety of institutions taking either traditional labs or participating in a national semester- or year-long CRE (the Science Education Alliance-Phage Hunters Advancing Genomics and Evolutionary Science [SEA-PHAGES] CRE). The SEA-PHAGES program engages students from dozens of institutions in a research education community where students isolate and characterize bacteriophage (11). Students in our study showed significantly higher measures for science identity, scientific community values, and project ownership content than students in traditional labs (Fig. 3A). However, our students showed only marginally higher scores in self-efficacy, project ownership emotion, and networking than traditional lab students. In comparison to the students who participated in the SEA-PHAGES program, our students showed similar scores for measures of self-efficacy, science identity, and scientific community values but significantly lower scores for project ownership content, project ownership emotion, and networking (Fig. 3A). Hanauer et al. (20) also report scores for students from an institutional context more similar to that of our students (R1 universities). Figure 3B compares data from our students to a data set from Hanauer et al. for a subset of students from R1 institutions. In this comparison, we again found similar scores for self-efficacy and scientific identity and significantly low scores in project ownership (content), project ownership (emotion), and networking between our students and the SEA-PHAGES students. In contrast, the difference in scientific community values between our students and the SEA-PHAGES R1 subset was greater than when we compared our students to the entire sample of SEA-PHAGES students.

## DISCUSSION

Many semester-long CREs have students conduct research on a single research question throughout the semester (9–11). In contrast, we have implemented three different SREs based on faculty research in our department. Student perceptions of learning gains from three diverse SREs suggest that all were valued by students and perceived as contributing to their learning. We found no evidence for increased perceptions of gains based on the length of the SRE. We also did not find evidence that the number of lab techniques employed or model systems impacted student perceptions of learning. Student perceptions also seemed unaffected by the novelty of their results as the *Candida* mutation rate module involved students performing technical replicate measurements of a biological sample, whereas the bean beetle microbiome and C. elegans modules had students analyze a unique beetle or C. elegans population.

Although we found no difference in student rankings between SREs, we did find consistent differences between rankings of competencies for biological experimentation across all three SREs. Students rated all modules as helping the most with conducting experiments, analyzing results, and drawing conclusions. All three modules gave students active instruction and practice doing these skills. Students ranked the modules as helping the least with understanding the primary literature, which is consistent with the lab curriculum that did not have any assignments or activities directly aimed at this skill. Observed differences in ratings between modules for “understanding primary literature” may reflect the timing of assignments more than module content as modules with earlier writing assignments (where students may reference the primary literature) scored higher than modules with later writing assignments.

We examined changes in three psychosocial measures (science identity, science self-efficacy, and scientific community values) from the beginning of the semester to the end of the semester. As this study was performed at a highly selective university, students already ranked themselves highly at the beginning of the semester, making increases difficult to detect due to ceiling effects. Average scores for individual items were all above 3.75 on a 5-point scale for self-efficacy and science identity, with most being over 4, and all were over 5 on a 6-point scale for scientific community values. Despite the high scores at the beginning of the semester, we found significant increases for all three measures from the beginning to the end of the semester, supporting the efficacy of our modular CRE model. Among the three measures, self-efficacy showed the greatest gains. Gains in self-efficacy may precede gains in medium-term outcomes such as science identity and community values (25, 26), resulting in lower gains for these measures.

Previous studies suggest that an SRE or a modular CRE does not have the same impact on student self-reported gains (15) or measures of student affect (16) as a semester-long CRE. However, students who participated in our introductory laboratory course with modular CREs had levels of several psychosocial measures similar to those of students who participated in the semester-long SEA-PHAGES CRE (20). In particular, scientific self-efficacy, scientific identity, and scientific community values for our students were similar to those observed in the semester-long CRE and significantly greater than those for traditional labs. Perhaps, combining several SREs or CRE modules within a semester increases the impact of these research experiences. In fact, Mader et al. (15) found that courses with a sequence of modules that included novel research questions and student involvement in experimental design led to student self-reported gains similar to those of semester-long CREs, whereas short CRE modules incorporated within a traditional lab course resulted in lower gains. In our course, student perceptions of project ownership (content) were intermediate between levels for traditional labs and those for the SEA-PHAGES CRE. The lower level of project ownership with modular CREs than with a full-semester CRE could be due to the shorter time period devoted to each project, where students could develop a sense of ownership over the project. Previous research suggests improved student outcomes with greater time investment in CREs (27).

For two psychosocial measures included in the PITS survey (19) (project ownership [emotion] and networking), students in our course were more similar to students in traditional laboratory courses than to those in the SEA-PHAGES CRE (20). Project ownership (emotion) measures the extent to which students perceived being delighted, happy, joyful, amazed, surprised, or astonished during their lab experience (19). Longer exposure to a single project as in a semester-long CRE might allow students to develop stronger emotional attachments to their research and hence more frequently experience these positive emotions in a full-semester lab. The lower levels of networking for students in our course than for students in the SEA-PHAGES CRE could be related to differences in project ownership. If students are more invested in their work and feel a greater sense of ownership over their work, they may be more likely to discuss their research with others (i.e., network). Alternatively, the structure of the SEA-PHAGES CRE could have resulted in students having a greater sense of networking. SEA-PHAGES is a national CRE, so it has a built-in network of students, educators, and researchers from a wider network than would exist in local SREs (11, 20). A comparison of students’ perceptions of networking in a local, semester-long CRE to those of networking in a local, modular CRE would help to elucidate potential explanations for this difference in perceptions of networking.

Although some outcome measures for students in our modular CRE were lower than those for students in the semester-long SEA-PHAGES CRE, these differences could be explained by differences in our samples. A much higher percentage of students in our course completed the survey than in the published data set for the SEA-PHAGES CRE (20) (93% and 52.2%, respectively). In addition, students in the SEA-PHAGES CRE generally self-selected to participate (15). Students with a strong investment in the research or course may be more likely to both invest the time to complete the survey and have positive perceptions of their lab experience, which could result in better student outcomes for students in the SEA-PHAGES CRE data set.

The modular SRE model that we implemented may have additional benefits not measured in our analyses. Lack of faculty time has been reported as a major impediment to CRE development (28). The development of short-term SREs is likely to be less time-intensive for research faculty than the development of entire-semester CREs. The inclusion of research from multiple faculty members in a semester course also broadens the number of faculty on a campus involved in undergraduate course-based research experiences and expands the breadth of research approaches that undergraduate students are exposed to through their lab courses. Modular SREs also allow a more stepwise progression from a traditional lab course to a research-based lab course and allow stepwise updates to the course rather than necessitating complete overhauls whenever research projects need to be changed. The use of several CRE modules also allows courses to cover different dimensions of course-based undergraduate research experiences (CUREs) (29) or different core competencies of scientific practice (1) across multiple modules, thus relieving pressure to design a single project that can accomplish all.

In addition to the benefits for students in our introductory biology lab course, faculty involved in our SRE modules reported positive outcomes for their research labs, such as increased recruitment of students (who enter with basic knowledge of their model system and specific lab techniques) and use as outreach for research grants. In fact, all three of the SRE modules developed as part of an introductory lab semester and presented here have since been expanded into educational projects funded by the National Science Foundation (DUE-1821533, DUE-1821184, DEB-1750553, and DEB-1943415). The beetle microbiome project led to an IUSE grant that trains others in the use of this system for a semester-long CRE (22), the C. elegans module was further developed into a semester-long upper-level lab course via a CAREER award, and the *Candida* project was expanded into an upper-level seminar course on writing about research via a CAREER award.

Overall, our results suggest that modular SREs can capture similar benefits of semester-long CREs for at least some measured outcomes. Given that modular SREs may reduce the burden on faculty time investment, offer increased curriculum flexibility, and allow exposure to multiple research approaches and fields, these results indicate that modular SREs may be a fruitful model for CRE implementation.
