# Supplementary material for: Assessment of Course-Based Research Modules Based on Faculty Research in Introductory Biology
Source: J Microbiol Biol Educ. 2021 Jul 30;22(2):e00148-21. doi: 10.1128/jmbe.00148-21 (PMC8442019; doi:10.1128/jmbe.00148-21)
Supplement: Supplemental file 1 — Supplemental material. Download JMBE00148-21_Supp_1_seq6.docx, DOCX file, 0.1 MB [file jmbe00148-21_supp_1_seq6.docx]

Supplementary Materials.

Appendix 1 – Module descriptions

*Bean beetle microbiome.* Students were given male or female bean beetles raised on mung beans (*Vigna radiata*), adzuki beans (*Vigna angularis*), or black-eyed peas (*Vigna unguiculata*). After sterilizing the outside of sacrificed beetles, students collected microbes in a saline solution from the interior of beetles and plated onto several types of growth medium (nutrient agar, EMB agar, PEA agar, and blood agar). Students collected colony morphology data of resulting bacterial colonies and calculated Inverse Simpson’s Diversity Index using colonies with distinct morphologies as separate taxa. In addition, each student selected a single colony to analyze by PCR of the 16s rRNA gene, Sanger sequencing, and BLAST analysis to identify bacterial genus and, if possible, species. Students further analyzed a mi-Seq dataset of high-throughput sequencing data of bacterial 16s DNA sequences collected from beetles raised on different diets. The data collected by students are added to online databases of colony phenotype and colony-based sequencing data that are available on the Bean Beetle website ([www.beanbeetles.org](http://www.beanbeetles.org)). These data are available for others to search and use in community analysis (1). Students were assessed during this module via lab notebook entries, homework assignments, weekly quizzes, multiple abstract and figure with caption assignments on lab-group and whole-class datasets, and a final exam with a practical component.

*Candida mutation rates*. The Hickman lab studies how eukaryotic pathogens generate genetic and phenotypic variation in response to drug or environmental stress. In this module, students were given cultures of *Candida albicans* strains with either haploid, diploid, triploid, or tetraploid genomes and single copies of a histidine synthesis gene. The histidine gene had a point mutation that made it non-functional so initial *Candida* strains were histidine auxotrophs. After growth in liquid media, students plated cultures onto nutrient-rich and histidine-deficient plates in order to measure revertant rate and calculate mutation rates for each strain. Students analyzed both their own data as well as the class-wide dataset. Students were assessed during this module via lab notebook entries, homework assignments, weekly quizzes, multiple abstract and figure with caption assignments on lab-group and whole-class datasets, and a final exam with a practical component.

*C. elegans evolution.* The Morran lab studies factors that facilitate or constrain adaptive evolution. Two lab-periods were devoted to this module along with a small amount of time in each lab throughout the semester that was used to passage the *C. elegans.*At the start of the semester, students were given a population of naïve *C. elegans*, which had not been exposed previously to pathogenic *Serratia marsescens* bacteria. Throughout the semester, students repeatedly passaged their population onto nematode growth medium with *Serratia marsescens* on one third of the plate and OP50 *E. coli* standard *C. elegans* lab food strain on the far third of the plate with the middle third of the plate without bacteria. Students collected surviving *C. elegans* from the OP50 side of the plate each week and re-passaged onto the middle of a new plate with pathogen and OP50 sides. They also did this same process for a control population of *C. elegans* that were passaged on plates where the *Serratia* had been heat-killed before plating. At the end of the semester, students assayed their *C. elegans* population for avoidance of and resistance to *Serratia.* Student analyzed both their own data as well as the class-wide dataset. Students were assessed during this module via lab notebook entries, homework assignments, weekly quizzes, multiple abstract and figure with caption assignments on lab-group and whole-class datasets, and a final exam with a practical component.

Appendix 2– Survey validation

We validated the PITS instrument on our student population by measuring internal consistency of each scale using Cronbach’s alpha. Alpha metrics for all scales in pre- and post-semester datasets were above 0.8, indicating validity of the instrument for our student population. We also validated the PITS survey on our student population using confirmatory factor analysis using the lavaan package in R (2).  The comparative fit indices (CFI) for the pre-semester and post-semester datasets were 0.94 and 0.86, respectively, with a value of 0.9 or greater suggesting a good fit of the model. For both datasets, the Root Mean Square Error of Approximation (RMSEA) were significant (P<0.001), suggesting that the models were significantly different from a close fit.  However, the RMSEA values were not large (0.07 and 0.08 for pre- and post-semester datasets, respectively, with a value of 0.05 or lower suggesting a good fit for the model). Yet, modification indices have the highest values for items that are in the same factor, which suggests that the lack of close fit is mainly due to differences in how items within a factor are correlated with one another. Overall, the confirmatory factor analysis supports the conclusion that the PITS instrument and underlying factors are valid for our student population.

Appendix 3– Individual item results


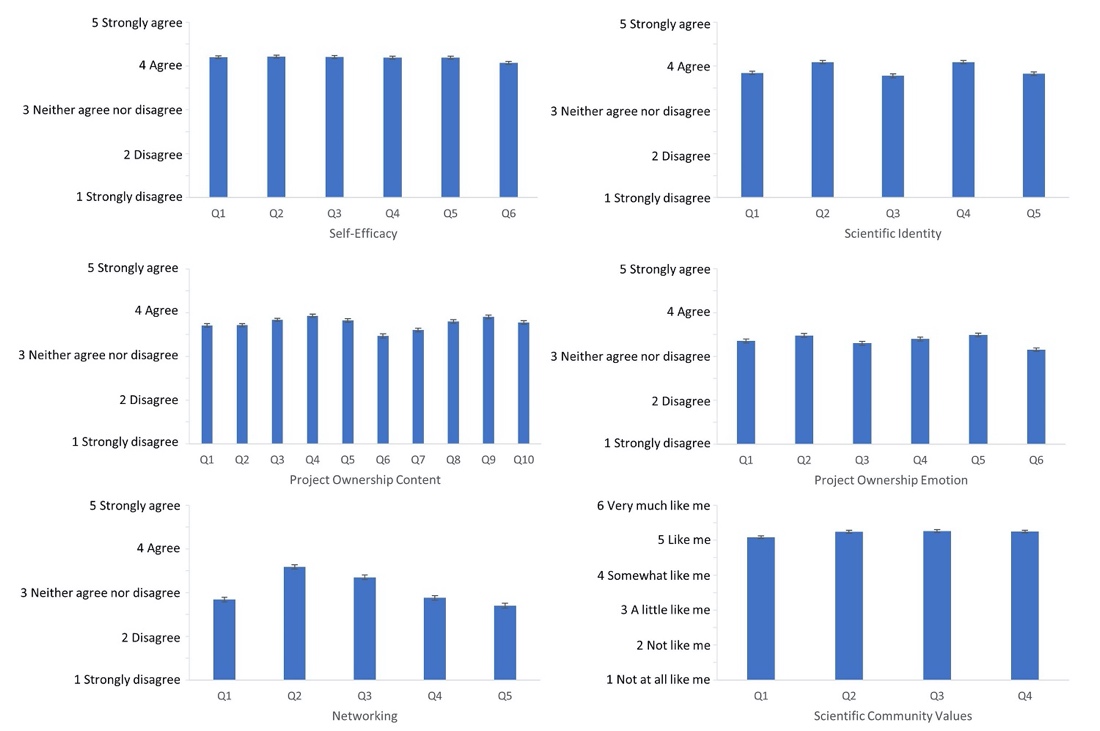


Figure S1. Individual item scores for post-semester survey. Average scores for individual items using 5-point (self-efficacy, scientific identity, project ownership content, project ownership emotion, and networking) or 6-point (scientific community values) Likert scales. Error bars are SEM.

Appendix 4 – Item prompts

|  | Prompt | Item |
| --- | --- | --- |
| Science Self Efficacy | Rate the degree to which you agree or disagree with the following statements concerning your confidence in your abilities to function as a scientist in your area. | I am confident that I can use technical science skills (use of tools, instruments, and techniques) |
|  |  | I am confident that I can generate a research question to answer |
|  |  | I am confident that I can figure out what data/observations to collect and how to collect them |
|  |  | I am confident that I can create explanations for the results of the study |
|  |  | I am confident that I can use scientific literature and reports to guide my research |
|  |  | I am confident that I can develop theories (integrate and coordinate results from multiple studies) |
| Science Identity | Rate the degree to which you agree or disagree with the following statements concerning your sense of yourself as a scientist who undertakes research activities: | I have a strong sense of belonging to the community of scientists |
|  |  | I derive great personal satisfaction from working on a team that is doing important research |
|  |  | I have come to think of myself as a 'scientist' |
|  |  | I feel like I belong in the field of science |
|  |  | The daily work of a scientist is appealing to me |
| Project Ownership Content | Rate the degree to which you agree or disagree with each statement: | My research will help to solve a problem in the world |
|  |  | My findings are important to the scientific community |
|  |  | I faced challenges that I managed to overcome in completing my research project |
|  |  | I was responsible for the outcomes of my research |
|  |  | The findings of my research project gave me a sense of personal achievement |
|  |  | I had a personal reason for choosing the research project I worked on |
|  |  | The research question I worked on was important to me |
|  |  | In conducting my research project, I actively sought advice and assistance |
|  |  | My research project was interesting |
|  |  | My research project was exciting |
| Project Ownership Emotion | In the table below you will find 5 different emotion words from a standardized emotion index. Think about the laboratory course you participated in and wherever relevant please indicate the extent to which each word describes your experience of that course: | Delighted |
|  |  | Happy |
|  |  | Joyful |
|  |  | Amazed |
|  |  | Surprised |
|  |  | Astonished |
| Networking | Rate the degree to which you agree or disagree with the following statements dealing with the discussion of your research: | I have discussed my research in this course with my parents (or guardian) |
|  |  | I have discussed my research in this course with my friends |
|  |  | I have discussed my research in this course with students who are not in my class but in my institution |
|  |  | I have discussed my research with students who are not at my institution |
|  |  | I have discussed my research in this course with professors other than my course instructor |
| Scientific Community Values | Please read each description and think about how much each person is or is not like you. Check the answer that best reflects how much the person in the description is like you: | A person who thinks discussing new theories and ideas between scientists is important |
|  |  | A person who thinks it is valuable to conduct research that builds the world's scientific knowledge |
|  |  | A person who thinks that scientific research can solve many of today's world challenges |
|  |  | A person who feels discovering something new in the sciences is thrilling |

Table S1. Individual item questions for post-semester survey. Items from Hanauer et. al. (3).

References:

1. Blumer L, Beck C. 2020. Introducing community ecology and data skills with the bean beetle microbiome project. Advances in Biology Laboratory Education 41:art24.

2. Rosseel Y. 2012. lavaan: An R Package for Structural Equation Modeling. Journal of Statistical Software 48:1-36.

3. Hanauer DI, Graham MJ, Hatfull GF. 2016. A Measure of College Student Persistence in the Sciences (PITS). CBE-Life Sciences Education 15:ar54.
